# Longitudinal qualitative study on the psychological experiences of COVID-19 patients based on timing it right framework

**DOI:** 10.1038/s41598-024-63215-4

**Published:** 2024-05-30

**Authors:** Liangyan Zhang, Chen Zhang, Kesang Li, Yan Zhang

**Affiliations:** 1 Hemtology Department, Ningbo No. 2 Hospital, Ningbo, Zhejiang China; 2 Intensive Care Unit, Ningbo No. 2 Hospital, Ningbo, Zhejiang China; 3 Infection Department, Ningbo No. 2 Hospital, Ningbo, Zhejiang China

**Keywords:** COVID-19, Longitudinal qualitative study, Timing it right framework, Psychology, Health care

## Abstract

Timing it right framework was used as a framework to explore the illness experiences of patients infected with COVID-19 and to analyze the patients' perceptions of the disease and their true inner feelings to provide a reference for the control of infectious diseases. This research adopted a phenomenological research approach to develop a longitudinal qualitative study. A purposive sampling method was used to select participants and 37 patients were recruited. Depending on the principle that participants should have maximum variation and sampling should cease when interviews content saturation is achieved, 16 COVID-19 patients in an isolation ward in Ningbo City, Zhejiang Province were finally included. Data were collected using semi-structured interviews, and the content of the interviews was analyzed by Colaizzi’s 7-step method. The themes of COVID-19 patients’ experiences at various phase were presented as follows: multiple emotions intertwined at the time of diagnosis (anxiety, stressful panic, facing the diagnosis calmly), multiple pressures during the hospitalization period (concerns about the disease, unable to adapt to the ward environment, worrying about future hardship), growth of positive illness experience during the isolation and observation period (sublimated outlook on life, affirmation of the government's anti-epidemic policy, more concerned about their own health), adjustment after returning to society (stigma, loss of previous living environment, problems caused by nucleic acid testing), and adaptation to social life (return to normal life, avoidance of illness experience, post-covid-19 syndrome). The illness experience of COVID-19 patients changed dynamically with time, but a sense of shame and uncertainty about recovery was present throughout the process. Interventions should be developed according to the needs of the patients at different times to inform subsequent optimization of care and management of infectious diseases.

## Introduction

The ongoing global epidemic of coronavirus disease 2019 (COVID-19) has had a significant impact on human society, primarily in terms of public health. As of 2 February 2023, the reported cumulative number of confirmed cases worldwide is approximately 753 million, with approximately 6,814,900 deaths^1^. Importantly, the actual number of infections worldwide may be higher than the reported data due to factors such as limited testing capacity. The Omicron and Delta strains are variants of coronaviruses with high loads, high infectivity, short incubation times, and antigenic escape^2^. Patients experience long viral nucleic acid conversion times and are more likely to develop severe and critical illnesses, with long periods of hospital isolation and medical observation^3^. Studies have shown^4^ that patients with COVID-19 who stay in isolation are prone to psychological problems such as anxiety, depression, and fear. The study^5^ showed that infected patients had problems such as fatigue, sleep difficulties, and anxiety after discharge from the hospital, and those with severe lung damage during hospitalization were even more likely to be in the target population for long-term rehabilitation interventions after discharge; however, the study was conducted on patients at the beginning of the 2019 outbreak, and the characteristics of the coronavirus variants can cause various psychological experiences in infected patients. There are currently few studies exploring mild COVID-19 patients' experiences during hospitalization and after reintegration into society^6^. The Timing it Right (TIR) framework was proposed by Cameron et al.^7^ in the study of stroke care; it divides the process of disease development into five phases: diagnosis, stabilization, preparation, implementation and adaptation. At present, a number of scholars have used different research methods to study the dynamic needs of patients and caregivers with the TIR framework, and have achieved initial results^8^. Based on the TIR framework, it is possible to understand the changes in the experience of COVID-19 patients at different stages of illness, and formulate corresponding support plans, so that the intervention timing and intervention content can match the needs of patients at the current phase of disease^9,10^. Therefore, based on the five phases of disease development in the TIR framework, this research explore the illness experiences of COVID-19 patients in a longitudinal study to provide a reference for the subsequent care of patients with infectious diseases and the development of epidemic prevention and control strategies.

## Methods

### Design and participants

This study utilized a phenomenological research approach to develop a longitudinal qualitative study using the TIR framework. To conduct a longitudinal qualitative study, it is necessary to select specific time points for data collection, and the chosen time frame should be adequate for observing changes in the phenomenon under investigation^11^. In this study, the time frame for data collection was established through two interviews. The first interview (T1) took place during the period when the infected individuals were isolated, and the second interview (T2) occurred 6 months after their reintegration into society. The study employed a purposive sampling method to select patients who had been diagnosed with COVID-19 and were admitted to an isolation ward in Ningbo City, Zhejiang Province, between January and October 2022. The inclusion criteria were as follows: (1) confirmed COVID-19 infection; (2) age above 18 years; and (3) voluntary participation in this study and providing informed consent. The exclusion criteria were as follows: (1) those who were seriously ill and unable to communicate and (2) those with serious psychological or cognitive dysfunction. Those who automatically withdrew or were lost to follow-up during the interview were dropped from the study. A total of 37 patients were recruited for the study, and the sample size of the study was determined based on information saturation of the interviewees^12^. The specificity of the sampling was measured by demographic characteristics and the Self-Rating Depression Scale (SDS)^13^. Sampling ceased when interviews content saturation was achieved. Finally, 16 cases of representative infected patients were included in the study, including patients with no depression (9 cases, 56%), suspected mild depression (1 case, 6%), and suspected moderate depression (6 cases, 37.5%), numbered "N1" to "N16". In this study, two in-depth interviews were conducted with the infected patients, one during their isolation period and another after 6 months of reintegrating into society. Notably, during the second interview, two of the participants exhibited evasive behavior. For example, one participant was very talkative in the first interview, and he was able to talk more about his true feelings and describe them in more details based on the experience of the illness. However, in the second interview, he spoke less and only answered yes, no or fine, showing an evasive attitude. In order to fully understand the thoughts of the participants and enhance the integrity and continuity of the study, after obtaining informed consent, the interviewer supplemented the interview content by contacting their relatives, and returned the transcript of the interview content to the participants for confirmation, so as to ensure the authenticity of the content. The basic information of the study participants is shown in Table 1.

**Table 1:** Basic information of the participants (N = 16).

Num	Sex	Age	Education	Job condition	Marital status	Infection in the family	Comorbid condition	SDS score
N1	Female	34	Junior	Unemployed	Married	4	No	48.75
N2	Female	30	Junior	Clerk	Married	0	No	55.00
N3	Female	43	Junior	Clerk	Married	0	No	52.50
N4	Male	20	Junior	Freelancer	Single	0	No	60.00
N5	Male	34	College	Clerk	Married	4	HBV	66.25
N6	Male	41	College	Manager	Married	2	No	37.50
N7	Male	31	Junior	Worker	Single	0	HBV	50.00
N8	Male	25	Junior	Worker	Single	0	No	36.25
N9	Male	48	Primary	Worker	Married	0	No	70.00
N10	Male	38	Junior	Worker	Married	0	No	67.50
N11	Male	30	Senior	Clerk	Married	1	No	19.00
N12	Female	37	Junior	Worker	Divorce	0	No	42.50
N13	Male	47	Junior	Worker	Married	0	No	35.00
N14	Male	40	Junior	Worker	Married	0	HBV	60.00
N15	Female	30	Primary	Worker	Married	1	Epilepsy	65.00
N16	Female	39	Junior	Worker	Married	1	No	36.25

Different education and job condition may lead to differences in their Abbreviations understanding and acceptance of the disease.

### Data collection

The participants provided informed consent and completed the demographic scale and SDS scale assessment before the interview. The interview outline was designed according to the five phases of diagnosis, stabilization, preparation, implementation and adaptation in the TIR framework. Longitudinal qualitative studies require researchers to use the insights gained from previous interviews to inform the focus of subsequent data collection^14^. The researcher preinterviewed two infected individuals prior to the formal interview and amended the interview outline. The interview outline was as follows. During Phase 1 (T1), the following questions were asked: ① How did you feel when you were diagnosed with COVID-19? ② What was your initial feeling after arriving at the isolation ward? ③ What are your needs and feelings during hospitalization? ④ How do you feel when you go to the quarantine point for medical observation? ⑤ How will the Spring Festival affect you? ⑥ If you are cured and returned to society, what expectations or concerns do you have? During Phase 2 (T2), the following questions were asked: ① What was your mood at the beginning of reintegration? Give an example of 1–2 things that were particularly impressive at that time. ② How do you feel now? How has your life changed? ③ Is there anything particularly troubling you at present? How do you solve these troubles? Do you have any hopes or suggestions? Due to the requirements of epidemic prevention and control, the interview was conducted by telephone, and the interview time was 15–60 min.

### Data analysis

The interview content was analyzed by Colaizzi’s 7-step method. The steps are as follows: (1) two researchers carefully read all the original data; (2) they independently identify significant statements that were repeated by multiple participants and were important and meaningful to the research question; (3) they code repetitive and meaningful views by temporarily "suspending" their assumptions and value judgments; (4) they gather ideas after coding to form the prototype of the theme; (5) they write a detailed typical original description; (6) they identify similar ideas and condense them into themes; and (7) they return the topic structure to the participants for verification. The results of the SDS questionnaire were descriptive statistical analyzed by SPSS 25.0.

### Quality control

SDS questionnaire collection: during hospitalization, a researcher issued and collected the questionnaire, explained the questions appropriately, and checked the completeness of the answers on the spot. (2) Interview data collection: all researchers in the study received professional training in qualitative research, and all interview data collection was completed by the first author (isolation ward nurse). The researcher kept in touch with the participants during the follow-up period to establish trust and friendly relationships. The researcher used the daily chat information as supplementary information. At the end of each interview, the researcher transcribed the audio-recorded information within 24 h and recorded the tone of voice, pitch change and other information of the participants. (3) Data analysis: data collection and analysis were carried out in parallel, with each of the 2 researchers listening to the audio-recordings and reading the textual information repeatedly, independently summarizing statements of significance, and summarizing the text of the statements. And the themes and subthemes extracted from the collected data were repeated to the participants by instant messaging software to further confirm whether they had the same feeling or experience and record any possible supplemental information. It was explained to the participants at the first interview that the preliminary results would be reported back to them for verification.

### Ethics

This study was reviewed and approved by the Ethics Committee of Ningbo No.2 Hospital (YJ-NBEY-KY-2022-102-01). All methods were performed in accordance with the relevant guidelines and regulations such as ethical standards of the institutional ethics committee and with the Declaration of Helsinki. All participants signed informed consent forms.

## Results

An interpretive understanding of the illness experiences of patients infected with COVID-19 was constructed in this study. The analysis yielded five categories and fifteen sub-categories (Table 2).

**Table 2:** The categories and sub-categories of the study.

Categories	Sub-categories
(1) Multiple emotions intertwined at the time of diagnosis	(1) Anxiety
	(2) Stressful panic
	(3) Facing the diagnosis calmly
(2) Multiple pressures during the hospitalization period	(1) Concerns about the disease
	(2) Unable to adapt to the ward environment
	(3) Worrying about future hardship
(3) Growth of positive illness experience during the isolation and observation period	(1) Sublimated outlook on life
	(2) Affirmation of the government's anti-epidemic policy
	(3) More concerned about their own health
(4) Adjustment after returning to society	(1) Stigma
	(2) Loss of previous living environment
	(3) Problems caused by nucleic acid testing
(5) Adaptation to social life	(1) Return to normal life
	(2) Avoidance of illness experience
	(3) Post-COVID-19 syndrome

### Theme 1: multiple emotions intertwined at the time of diagnosis

Due to different personality characteristics and understanding of the disease, infected patients showed the following emotional changes in the early stage of diagnosis.

1. Anxiety: since most infected patients learned about their infection suddenly, most of them had significant fidgeting, irritability, insomnia, and felt inexplicably nervous and worried when they were diagnosed or preparing for medical isolation. N5-T1: "I was so anxious when I was diagnosed that I felt like the sky was falling." N9-T1: "Since that time I have not been able to sleep well at night, I wake up after an hour of sleep." N2-T1: "I felt like it was going by so slowly and every day felt like years. Before the quarantine, I was worried that I wouldn't eat or sleep well, and that was true." Some of the infected patients felt upset when they were first diagnosed, as they received many calls for epidemiological investigations from various departments and organizations. N6-T1: "The initial phase has been very busy. I was the first to be infected, and there was a particular focus on me. At that time, my sleep was disturbed."
2. Stressful panic: the knowledge that there was no effective treatment, the uncertainty of the sequelae and the possibility of recurrence all put the infected patients in fear of the disease at the time of diagnosis, with three infected patients displaying more pronounced despair. N12-T1: "At that time, I was lying in bed for two days feeling like I was going to die. It was bad anyway. I was scared, scared of the after-effects, scared for my life (emotional)." N9-T1: "[Messages on my mobile phone] made me think nonsense. If the virus in my body had stayed, I would have been locked up forever. It gave me a feeling of panic."
3. Facing the diagnosis calmly: some of the infected patients were not so afraid of the diagnosis because people around them had already been infected. N1-T1: "The mood was a bit panicky, not particularly panicky. Because my in-laws have already been diagnosed, I already have a general idea in my mind." Some infected patients had a more comprehensive understanding of the corresponding symptoms of infection and were more receptive. N11-T1: "Not afraid of the disease. I have read the news that most of the foreign countries are infected, so I am calm." N3-T1: "It was mild… and curable, so I just didn't have much stress anyway."

### Theme 2: multiple pressures during the hospitalization period

When patients were hospitalized, the disease itself, positivity for 2019-nCoV nucleic acid, and the specificity of the isolation environment made them feel deeply stressed.

1. Concerns about the disease: the threat to COVID-19 patients' lives and safety during hospitalization was stressful for those infected, either because of their own experiences of repositivity or because they witnessed others experiencing repositivity. N11-T1: "The biggest concern was that I didn't know how long it would take to be discharged from the hospital, how long it would take to recover … I didn't know if there were any after effects and whether there would be a relapse." N5-T1: "(After being repositive for the third time) I cried all the time during those 2 days when I was isolated again. The last 2 days were fine, (my mood) calmed down, and today I'm a little bit annoyed again, my wife and my youngest daughter they don't seem to have a good nucleic acid result. That aspect is affecting me a lot, a lot of stress (speaking faster)." N14-T1: "There is a feeling of fear in my heart. This is the first time I've had a repositive, and I've heard that there are a lot of repositive people, and I'm scared in my heart." Due to the special nature of COVID-19 patients, the vast majority of them chose to conceal their condition to avoid worrying about their families. N3-T1: "I didn't dare to make a video call to my mother. I was afraid that if my mother knew I was inside the hospital, she would have to worry about me."
2. Unable to adapt to the ward environment: most infected patients were transferred to isolation wards for medical observation and treatment, and the unfamiliar and confined environment often made them feel uncomfortable. N4-T1: "It feels unreal to be here, the air is treated and the rooms are airtight." Meanwhile, due to the shortage of beds, most isolation wards housed many people, to which some infected patients are not accustomed, and some infected patients were deeply stressed about secondary infections. N2-T2: "I don't like to sleep with other people in the same room. But, since I was a child, I have been timid and I am afraid to sleep alone. I am not used to sleeping in the hospital." N12-T1: "I was worried about my problems and whether sleeping in the same ward would spread the virus to each other, so I never took off my mask." Isolation also prevents infected patients from spending time with their families. During the festive season, which symbolizes reunion, they had to spend time alone. N10-T1: "The arrival of Chinese New Year had an impact on me. There are so many things I can't do because (I can't) be reunited with my family."
3. Worrying about future hardship: because the whole family was quarantined or because they themselves were the main laborer in the family, most infected patients reported that the long period of isolation had affected their financial income and were worried about the future. N5-T1: "There has been no income for a few months now, which means that life may be hard in the future." Due to the release of information from epidemiological surveys, infected patients are often worried about having the disease known to their acquaintances. N5-T2: "[The flow survey information] would be better to change that column where the surname is written to a number. Because we are the only ones infected in the factory, it is easy for others to guess." Infectious patients worry about social discrimination brought about by the disease and about whether they will still be able to have a suitable learning environment, job, and residence in the future. N5-T1: "I worry about whether it will affect my youngest daughter's ability to go to a better kindergarten." N10-T1: "I feel that I will not be able to find a job if people know about my disease. … I am sure I will be treated differently." "N2-T1: "We are renting a house outside, the landlord knows about it and may not let us stay in this place. What should we do?"

### Theme 3: growth of positive illness experience during the isolation and observation period

Patients were under medical isolation and observation during this period. There is deeper thinking after enduring pressure from all sides, and some patients gain growth.

1. Sublimated outlook on life. Some infected patients were grateful for the dedication of healthcare workers. N1-T1: "Really, I feel that you are all very good. Doctors have it so hard. I didn't feel it before." Some patients thanked the teachers for caring for their children during the infection. N1-T2: "The teachers and classmates are very caring for my daughter. In particular, her class teacher was really nice. There was no one to look after her when she first came out (family members were quarantined for infection). My daughter was alone at home, and I was worried. Her teacher let my daughter go to her house. The teacher said I am not afraid." Some infected patients also said that during the isolation period, they had time to calm down and question their souls and thought more about life. N6-T1: "Actually, when I was in the isolation ward, I had more time and space to think, and then I might think more deeply about something, like the meaning of life. I would find so many things that I just naturally realized."
2. Affirmation of the government's anti-epidemic policy. N6-T1: "I think our government departments are very committed because our national situation is different from that of foreign countries, with a large population. If our country really doesn't care about controlling the epidemic at all, the medical resources can't keep up." N9-T1: "I fully comply with the rules and regulations of the isolation ward. The country invests a lot of resources. We eat and live medically for free."
3. More concerned about their own health. The experience of falling ill has made more patients pay more attention to their own health, and they actively participate in sports and pay attention to physical checkups and rest. N11-T1: "It's better to have a medical checkup to see if this virus has any other effects on your body. In the future, I will have regular medical check-ups once every six months or a year. Now, I also started to run (during the quarantine phase), I didn't run much before."

### Theme 4: adjustment after returning to society

The infected often return to society in a state of fear and with a deep sense of stigma. Some infected patients have lost their former jobs and living environments, and their finances and lives have been greatly affected. At the same time, the nucleic acid test also brought troubles to their lives after returning to society.

1. Stigma: infected patients often have a heavy self-psychological burden when they return to society. They are afraid of being rejected and treated differently. The main manifestations are fear of stigma and concealment of medical history. N1-T2: "We definitely have to report to the community when we go back. We have to explain the situation. After explaining the situation, the people in the village will be scared to death, they will ostracize them (brother, parents), and then it will definitely have an impact on my parents and my brother's lives, so we didn't dare to go back." Some infected patients were worried that they would cause others to become infected, causing them to be quarantined as well. N16-T1: "I'm worried that if I go back to being positive in the future, I will infect others, even if it doesn't hurt, will I have to quarantine people for so many days?" With the shame of the disease, many infected patients were unable to go home to their families. N1-T2: "I would love to go back to my hometown. Because my parents are also 70 years old, and I can't take care of them when I'm out of town. But, now I can't go back to my hometown."
2. Loss of previous living environment. Some infected patients lost their original living and working environments after returning to society. Some COVID-19 patients moved away from their original environments of their own initiative because they were afraid that others would know that they had been infected by COVID-19. N1-T2: "We moved home. But, we are still afraid of bumping into people we know: "Some infected patients were asked not to go to work or to change their working environments for fear of mutual infection in their workplaces. N2-T2: "We were not allowed to go to work in the factory at that time because the other employees didn't agree to let us go to work." N9-T2: "She used to work in Department 5, but the original department didn't want her anymore." A new environment means a new start, which leads to greater pressure on the lives of infected patients. N1-T2: The pressure on my husband's life is too great. I also went to look for a job. After looking for a few days, I felt very sad and found that I could only do more flexible work like takeaway. Life is difficult, we've borrowed a lot of money now, and there's a lot of financial pressure and mental stress."
3. Problems caused by nucleic acid testing. Because of previous infections, policies require infected patients to have a separate nucleic acid test. This often meant that they had to spend time and effort looking for a separate site where they could do a separate nucleic acid test. N1-T2: "My child can't do a separate nucleic acid test at school anyway, so we have to find a nucleic acid test site by ourselves." When doing the nucleic acid test alone, they were split into two teams, separate from the people doing the mixed nucleic acid test, and the people in their team were faced with more inquiries about infection, which put more pressure on them." N1-T2: "When we went to do the nucleic acid test, the security guard at the door asked once, then the person who checked the identity after the questioning asked again, and the sampler asked again. I had nightmares every day." In addition, they were concerned that doing the nucleic acid test alone would increase the risk of reinfection. N5-T2: "When we do the nucleic acid test alone, we are with people who are at high risk. The book they have registered in we touch it again, register it again and go with them. If I get infected again, I reckon I'm going to go mad." Because of the fear of repositivity, infected patients are extremely concerned about the results of their own and their family's nucleic acid tests." N3-T2: "To be honest, some people, like my wife, have repeated repositive and go to quarantine sites over and over again. Very upset and depressed. I'm worried about my own and my wife's nucleic acid test results."

### Theme 5: adaptation to social life

Six months after returning to society, most of the infected patients returned to normal life with the support and tolerance of society, but some of them still avoided infection and concealed their status as recovered persons. Some of the infected patients developed post-COVID-19 syndrome.

1. Return to normal life. Some infected patients integrated into society with the tolerance of colleagues and friends around them. N2-T2: "Still working as normal. My friends and I still play well and sit together for meals." N8-T2: "I was a bit afraid that my colleagues would laugh at me in various ways before I came to work. But, none of them did." Nucleic acid testing is also gradually becoming less frequent. N7-T2: "In the beginning, it was a single test, now it's all mixed." Some infected patients said that they no longer avoided the fact that they were infected under self-regulation and gradually returned to normal life. T12-N2: "When I first came out of quarantine, whenever people talked about this stuff, I wanted to avoid it and didn't want to hear about it. Now, when people talk about doing nucleic acids, I seem to slowly be able to accept it."
2. Avoidance of illness experience. Some infected patients still avoided infection and concealed their identities as a recovered person. N16-T2 Husband added: "Whoever mentions this, she is anxious about it. Some old folks called me and asked about this, and she was also a little anxious in her heart. There is nervousness in her heart, and she doesn't want to talk about it anymore." During the interview with N5-T2, the infected patient herself said, "I don't feel anything, it doesn't affect me at the moment. Everything is fine. Don't want to think about it." However, interviewing his lover, he said, "He used to look like a child, he used to laugh and joke around, but now he looks sad every day. He doesn't talk much in his free time." This experience has changed his personality.
3. Post-COVID-19 syndrome. Some infected patients still have residual weakness, chest tightness, loss of smell, sleep disorders, etc. N14-T2: "The body is a bit different anyway. I'm more tired than I used to be at work, and I'm not as fit as I used to be. Sleep has been bad, easy to wake up. I have no sense of smell." N15-T2: "I feel a bit breathless sometimes when I walk a few steps. I feel tired."

## Discussion

This study conducted a longitudinal exploration of COVID-19 patients' experience based on the TIR framework. In general, the TIR framework has played a good guiding role in the exploration of COVID-19 patients' illness experience. During the diagnosis phase, COVID-19 patients were usually very concerned about their own health problems, and due to great pressure caused by isolation, they were prone to psychological problems. The stabilization and preparation phase of COVID-19 patients were in the long isolation treatment. Patients gradually adapted to isolation treatment and started to plan their life after discharge. They were worried about discrimination and worried that they would never go back to the original life. In addition, because COVID-19 patients were kept in isolation for a long time, some patients began to reflect on the experience of the disease and the past life status, and thus gained growth. The stigma of COVID-19 patients was felt throughout the course of the illness, but it was particularly evident during the implementation and adaptation phase. Due to the stigma and discrimination, some patients actually made great changes to their work and life during this two phase. At this time, social support was particularly important for patients to reintegrate into society. The following discussion provides detailed analysis of the above phenomena.

The results of this study showed that all infected patients diagnosed for the first time had psychological problems such as anxiety, fear, and sleep disorders, and seven patients (43.75%) were suspected of having mild to moderate depression according to the SDS scores, which is similar to the results of the study by Deng et al.^15^. This may be due to the highly contagious nature of COVID-19 and the lack of specific treatment, resulting in infected patients being prone to excessive stress, panic, and even psychological stress disorder at the early stage of diagnosis^16^. At the same time, 14 cases (87.5%) of the infected patients expressed concern about the possibility of "repositivity" after recovery and the existence of sequelae. A related study found that noninfectious viral RNA persisted in most of the "repositive" cases, which may be due to slow disease regression^17^. A positive test does not always mean that the patient is infectious, as it may detect fragments of viral nucleic acid^18^. However, most patients know little about this and develop more anxiety and fear. In contrast, those patients who have knowledge of the new coronavirus tend to have less fear and worry. Cheng Hualing et al.^19^ meta-integrated the psychological experience of COVID-19 patients in China and found that more information provided by medical personnel to infected patients about the treatment and prognosis of the disease could eliminate the anxiety caused by the patients' lack of knowledge. Therefore, health promotion should be strengthened, and patients should be encouraged to acquire knowledge of the disease in various aspects, including transmission channels, protective measures, symptom classification, prognosis, etc. Patients’ questions should be patiently answered based on their own conditions, and they should be encouraged to obtain correct information through official authoritative channels to avoid "information panic"^20^.

Four infected patients (25%) in this study indicated that the relatively confined environment and instruments in the isolation area caused feelings of loneliness and helplessness. The isolation treatment measures separated the patients from the outside world, as they left their familiar working and living environments to enter a state of isolation, confinement and monotony. The patients' original lifestyles were completely disrupted, and certain financial losses were incurred. The unfamiliar environment and medical equipment increased the patients' fear and loneliness while also hindering the discharge of their negative emotions. This is consistent with the findings of Wang et al.^20^ and Shaban et al.^21^. A retrospective study^22^ found that isolation may cause unexpected mental trauma for patients and may even lead to self-injurious behaviors such as suicide. These effects persisted 3 years after desegregation. Psychological disorders can lead to low immunity and reduced motivation for treatment, thus negatively affecting disease recovery^23^. Currently, the treatment and care of patients in isolation wards are mainly focused on the disease itself, with relatively little attention given to the physical and psychological effects of isolation. Improving the treatment environment in isolation areas, providing as much stimulation as possible during the normal routine of work and rest, establishing a good lifestyle, and diversifying hospital activities are of great significance in relieving emotional tension, breaking psychological barriers, and promoting disease recovery^24^. Studies have shown that having sufficient sunlight is particularly effective for psychological relief^25^. Isolation areas should be set up to face south, and lighting measures should be strengthened. Public corridors can be set up in the isolation area so that infected patients can stagger their activities appropriately. Healthcare workers who are fully "armed" can paste their names on their protective clothing to improve communication between doctors and patients and reduce patients' sense of isolation. There are also studies^26^ suggesting the use of the Rosenthal effect, that is, using praise, trust and expectation, and other psychological hints to help patients regain confidence and obtain positive motivation to change their own behaviors. By communicating with patients to understand their psychological needs, sources of negative emotions and specific factors affecting their emotions, healthcare professionals, in collaboration with teams from various disciplines, can encourage infected patients to cope positively and provide social support and psychological guidance to improve their quality of life^27^. The patients in this study were often in a state of confusion and worry before discharge and were also often unable to adjust to a good rhythm of life within a short period after discharge. Healthcare professionals can help patients develop a postdischarge transition plan during inpatient isolation so that they can quickly return to their original lives. At the same time, a scale of social reintegration behavior of infected patients with infectious diseases that is suitable for our country should be developed to prospectively investigate the current status of social reintegration of infected patients and to understand the changes in social reintegration behaviors in different periods to take corresponding measures to help such people adapt to their situations as soon as possible.

As a contagious disease, COVID-19 can lead to fear among the population as well as stigma and discrimination against specific groups of people^28^, and patients develop a sense of shame about their illness as a result. The results of this study show that patients often want to keep their experience of the disease confidential. The vast majority of infected patients in this study chose to conceal their illness from their family members. Due to the existence of a sense of shame, patients not only kept their disease experience secret but also often consciously reduced their mobility after discharge from the hospital to avoid infecting others. Patients may actively distance themselves from their friends and relatives, resulting in the loss of an important source of social support and a sense of isolation^29^. There were also patients who did not actively conceal their condition, but friends and colleagues of the infected patients intentionally chose to distance themselves from the infected patients out of fear and rejection after learning of their condition. Six patients (37.5%) in this study were thus forced to leave their original places of residence or work, causing them to develop more negative emotions. On the other hand, the infected patients who indicated that their colleagues and friends treated them no differently than before reported that they resumed normal life more quickly after returning to society. Yuan et al.^19^ showed that the social support system of infected patients is an important factor in their posttraumatic growth. Social support based on kinship is the main way for most Chinese people to obtain social support. If this basic relationship is damaged, it prevents infected patients from obtaining the understanding and support of others, which can have a great impact on their physical and mental health^30^. The establishment of a good social support system will enhance psychological health; in contrast, the accumulation of negative emotions will lead to a variety of psychological problems^31^. Healthcare workers in the diagnosis and treatment of COVID-19 patients not only need to give the necessary treatment measures but also need to comprehensively assess the degree of understanding of the disease and social support system of infected patients. Healthcare workers should encourage infected patients to inform their families of their illnesses through daily communication, collaborate with their families to provide relevant psychological care, and improve the level of posttraumatic growth of patients^32^. In addition, the limitations of the public's knowledge of the disease will increase the individual's self-psychological burden, which will deepen the self-perception and experience of public stigma, resulting in the internalization of stigma^33^. Therefore, it is necessary to strengthen the information dissemination of infectious diseases, set up relevant policies for social groups such as communities and companies to avoid the public's rough treatment of infected patients returning to society, to protect the normal work and lives of infected patients and to reduce economic losses. This study shows that the policy benefits given by the government make infected patients believe in the national epidemic prevention policy and thus have confidence in the diagnosis and treatment of the disease. Therefore, state policy supports the reintegration of COVID-19 patients into society through macrocontrol.

Six months after returning to the community, some of the patients in this study still had sequelae of COVID-19, such as malaise, insomnia, chest tightness, and loss of smell. A recent study published in The Lancet^34^ also confirmed this phenomenon. Similar studies have shown that the acute phase of COVID-19 and subsequent health damage involves multiple systems, such as the respiratory, neurological, and cardiovascular systems^18^. Given that infected patients at this stage need professional guidance to avoid delaying their illness, we call for greater collaboration among scholars from different countries to share experiences in the treatment of the disease to improve the physical and mental health of the population in the face of the postinfection syndrome caused by the global pandemic of COVID-19 patients.

This study found that there is a lack of clarity in the division of labor between departments and duplication of investigations by various departments in the process of epidemiological investigation.. It is recommended that the relevant departments should strengthen the integration and sharing of information by using big data and increase training in epidemiological investigation to improve efficiency^26^. Knowing the benefits of epidemiological investigation and personal information protection can reduce the uneasiness of infected patients, it is recommended that the media increase the scientific knowledge of epidemiological investigations and, at the same time, hide patients’ last names in the publication of epidemiological investigation information to protect the privacy of infected patients, and prohibit malicious human searches and other behaviors.

## Limitations

While the study has its merits, it also has its limitations. First, this study interviewed COVID-19 patients only in Ningbo and did not include patients from multiple regions and centers. Second, this study was conducted during the control phase of the epidemic in China, and except for filling out the SDS scale, which was face-to-face, both interviews were conducted over the phone, and preventing the use of visual aids.

## Conclusions

The objective of this study was to explore the perceptions and comprehension of patients infected with COVID-19 during the Chinese new coronavirus epidemic. COVID-19 patients encounter numerous psychological challenges while simultaneously experiencing physical discomfort, isolation, a sense of shame, and uncertainty regarding recovery. While some patients eventually adapt to their circumstances, not all are able to do so. Therefore, it is crucial for healthcare providers and families to provide support in order to facilitate patient adjustment to normal life. Interventions should be tailored according to the specific needs of patients at different stages, informing subsequent optimization of care and management strategies for infectious diseases.

## Data Availability

The datasets generated and analysed during the current study are not publicly available due privacy protection but are available from the corresponding author on reasonable request.

## References

[1] World health organization (WHO). Coronavirus (COVID-19) dashboard [DB/OL] . https://covid19.who.int/. 2023-02-03.

[2] Chen WX, Chen M, Zhou SS (2021). Comparison of three experimental methods for detecting antibody levels after immunization with COVID-19 vaccine [J]. Chin. J. Microbiol. Immunol..

[3] Zhou, X. Y., Wang, H., & Hang, H. R., *et al.* Epidemiological and clinical characteristics of SARS-CoV-2 Delta variant infected patients with COVID-19 in Gansu province [J/OL]. *Nurs. J. Chin PLA* 1–14 (2022).

[4] Chen, H. L., & He, Q. A meta synthesis of qualitative research on the psychological experience of patients with COVID-19 infection in China. *J. Southw. Jiaotong Univ.* **2022**(06), 73–82 (2023). http://kns.cnki.net/kcms/detail/51.1586.c.20221123.1638.018.html.

[5] Hanson SW, Abbafati C, Aerts JG (2022). Global burden of disease long COVID collaborators; Estimated global proportions of individuals with persistent fatigue, cognitive, and respiratory symptom clusters following symptomatic COVID-19 in 2020 and 2021 [J]. JAMA..

[6] Guo M, Kong M, Shi W, Wang M, Yang H (2022). Listening to COVID-19 survivors: What they need after early discharge from hospital—A qualitative study [J]. Int. J. Qual. Stud. Health Well-being..

[7] Cameron JI, Gigrac MA (2008). “Timing It Right”: A conceptual framework for addressing the support needs of family caregivers to stroke survivors from the hospital to the home [J]. Patient Educ. Couns..

[8] Liang LL, Zhang Y, Shi Y (2016). Research progress on application of timing theory in disease nursing [J]. Chin. Nurs. Res..

[9] Chu HL, Zhou YX, Ni KW (2021). Sample size was determined for qualitative interviews based on the information efficacy model [J]. Chin. Gen. Pract..

[10] Ding X, Yao J (2020). Peer education intervention on adolescents' anxiety, depression, and sleep disorder during the COVID-19 pandemic [J]. Psychiatr. Danub..

[11] Balmer DF, Richards BF (2022). Conducting qualitative research through time: how might theory be useful in longitudinal qualitative research? [J]. Adv. Health Sci. Educ. Theory Pract..

[12] Audulv Å, Hall EOC, Kneck Å (2022). Qualitative longitudinal research in health research: A method study [J]. BMC Med. Res. Methodol..

[13] Sun J, Qiu WJ, Li HY (2021). Psychological anxiety of patients with novel pneumonia during isolation period [J]. Hosp. Admin. J. Chin. People's Liberation Army.

[14] Zhang W, Jiang HJ, Lu WH (2020). To summarize the experience of psychological intervention and rehabilitation of patients with COVID-19 in mobile cabin hospital [J]. Chin. J. Nurs..

[15] Deng CY, Wang X, Li J (2022). To analyze the depression level and its influencing factors in patients with COVID-19 [J]. Tianjin J. Nurs..

[16] Yao LY, Wu WL, Zhang YC (2008). To evaluate the mental health status and nursing of 45 patients injured in earthquake [J]. Chin. J. Nurs..

[17] Gidari A, Nofri M, Saccarelli L (2021). Is recurrence possible in coronavirus disease 2019 (COVID-19)? Case series and systematic review of literature [J]. Eur. J. Clin. Microbiol. Infect. Dis..

[18] Chen H, Wen X, Zhou J (2020). To explore the effects of life intervention on anxiety, depression and quality of life in patients with COVID-19 [J]. J. Nurs. Sci..

[19] Yuan ZF, Shen RH, Liu SY (2020). Current status and influencing factors of post-traumatic growth in patients with COVID-19 [J]. Chin. J. Nurs..

[20] Wang JY, Wei LL, Wang G (2020). A qualitative study on disease experience of 15 patients with COVID-19 [J]. J. Nurs..

[21] Shaban RZ, Nahidi S, Sotomayor-Castillo C (2020). SARS-CoV-2 infection and COVID-19: The lived experience and perceptions of patients in isolation and care in an Australian healthcare setting [J]. Am. J. Infect. Control..

[22] Brooks SK, Webster RK, Smith LE (2020). The psychological impact of quarantine and how to reduce it:rapid review of the evidence [J]. Lancet.

[23] Li S, Zhang XL (2019). To analyze the effect of psychological nursing on elderly patients with chronic obstructive pulmonary disease [J]. China Rural Health.

[24] Chen JG, Tan XD, Zhang L (2020). Study on the influencing factors of psychological status of confirmed COVID-19 patients and quarantine visitors [J]. J. Nurs. Admin..

[25] Korkut S (2022). Research of the coronavirus anxiety, post-traumatic stress, generalized anxiety disorder, quality of life, and stress coping styles in COVID-19 survivor [J]. Psychol. Rep..

[26] Zhang SS, Xie WY (2020). To explore the effect of psychological nursing based on Rosenthal effect on negative emotions of patients with suspected COVID-19 [J]. J. Nurs. Sci..

[27] Yuan K, Huang XL, Yan W (2022). A systematic review and meta-analysis on the prevalence of stigma in infectious diseases, including COVID-19: a call to action [J]. Mol. Psychiatry..

[28] Huang LX, Li X, Gu XY (2022). Health outcomes in people 2 years after surviving hospitalization with COVID-19: A longitudinal cohort study [J]. Lancet Respir. Med..

[29] Wang T, Nong F, Yang L (2020). Current status and influencing factors of stigma in patients after COVID-19 recovery [J]. Shanghai Nurs..

[30] Zhen, X. Y. Quality of life and related psychosocial factors in people living with HIV/AIDS [D] (Shanxi Medical University, 2011).

[31] Zhou SJ, Huang XB, Qian Y (2020). Common psychological conflicts and coping strategies of COVID-19 patients [J]. Chin. Mental Health J..

[32] Nan FF, Zhao Y, Fu SF (2021). Remote psychological crisis intervention service under the COVID-19 epidemic in Zhejiang province [J]. China J. Health Psychol..

[33] Li RJ, Yang JP, Huu XQ (2022). Qualitative research on psychological experience of pregnant women with COVID-19 [J]. Milit. Nurs..

[34] Huang L, Yao Q, Gu X (2021). 1-year outcomes in hospital survivors with COVID-19: A longitudinal cohort study [J]. Lancet..

